# Diagnostic performance and confidence of an optimized deep-learning algorithm for the detection of intracranial hemorrhages

**DOI:** 10.1186/s13244-026-02249-w

**Published:** 2026-03-17

**Authors:** Franziska Tombach, Kristina Krompaß, Jan-Lucca Hennes, Anna Weber, Jan-Peter Grunz, Henner Huflage, Thorsten Alexander Bley, Julius Frederik Heidenreich, Philipp Gruschwitz

**Affiliations:** Department of Diagnostic and Interventional Radiology, University Hospital of Würzburg, Würzburg, Germany

**Keywords:** Intracranial hemorrhage, Computed tomography, Artificial intelligence, AI software, Machine supervised reporting

## Abstract

**Objectives:**

To evaluate the performance of an optimized deep-learning-based algorithm (AI) for the detection and subtyping of intracranial hemorrhage (ICH) in non-contrast cranial CT (cCT).

**Materials and methods:**

CCTs performed between 2020 and 2022 were processed using a pre-trained 3D-neural-AI to detect ICH. The current version was compared to the initial version (ICH) and the radiological report (ICH, subtypes) regarding diagnostic accuracy. A consensus of the radiological report and an additional reading by a radiologist (7 years of experience) served as the ground truth. We investigated the AI-generated confidence score as a threshold for clinical usage.

**Results:**

In the total cohort of 2960 cCTs (ICH prevalence 10.5%), the current AI prototype detected ICH with high sensitivity (93.9%) and specificity (96.1%). This resulted in an accuracy of 95.9% and a negative predictive value (NPV) of 99.3%, including 12 ICH-positive cases that were initially missed by the interpreting radiologists. Subtyping results were comparable between the AI and the radiologists. In the cohort processed with both prototypes (*n* = 996), the results of the current AI were slightly lower (sensitivity 88.3%; accuracy 94.4%; NPV 98.6%), yet it still outperformed those of the initial version (sensitivity 77.7%, accuracy 95.5%, NPV 97.4%), resulting in eight fewer false negatives and eleven additional true positives. A confidence score of 60% was considered a useful threshold, resulting in a significant increase of AUC (*p* = 0.018).

**Conclusions:**

The current AI algorithm achieves high diagnostic accuracy and negative predictive value. Combining AI-driven analysis with radiologists’ expertise may improve the overall performance and reduce the number of missed ICHs.

**Critical relevance statement:**

The dual use of AI as a control and triage tool can reduce radiology workload. Our results show AI reliably supports standardized exams with diagnostic quality comparable to radiologists, while transparent output enhances clinical acceptance and integration.

**Key Points:**

- Potentially life-threatening intracranial hemorrhages are time-critical and need accurate detection on non-contrast cranial CT.
- The optimized algorithm achieved high diagnostic accuracy for ICH detection similar to radiologists.
- The combination of radiologists and AI may improve the efficiency and diagnostic quality of ICH detection.

**Graphical Abstract:**

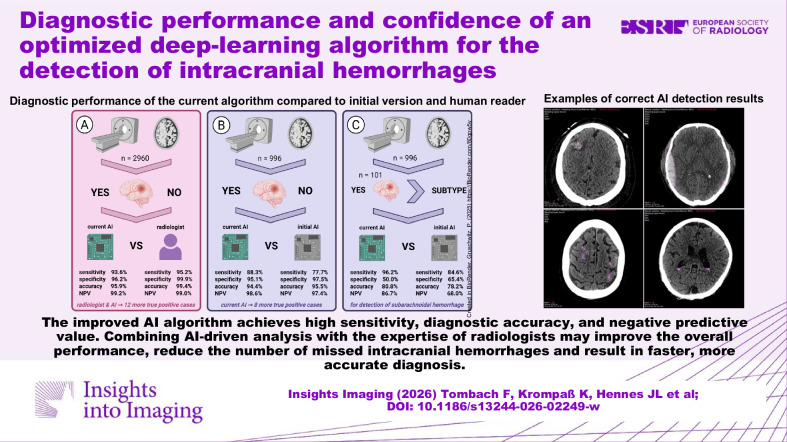

## Introduction

Intracranial hemorrhage (ICH) is a prevalent disorder, particularly among posttraumatic patients, with a prevalence of approximately 10% [1]. It has been demonstrated that ICH is associated with elevated morbidity and mortality rates of up to 40% within a 30-day period, particularly in cases where a diagnosis is delayed or therapeutic interventions are postponed [2]. Therefore, accurate and immediate diagnosis of ICH in emergency settings is essential within optimized clinical workflows. Cranial computed tomography (cCT) is the preferred method of choice for detecting intracranial hemorrhage (ICH) [3].

In the current medical context, radiologists are confronted with a substantial increase in the number of examinations performed on a daily basis. This increase is accompanied by a corresponding rise in the volume of images generated per scan, resulting in an escalating workload within the clinical routine. However, the number of radiologists does not increase proportionally [4]. Research has indicated a notable increase in CT cases during on-call hours (defined as nights, weekends, and holidays), with some studies reporting surges of up to 500% [5]. The visual focus of radiologists declines during the workday, leading to a significantly lowered diagnostic accuracy in a clinical setting [6]. Concurrently, the quality of reports from residents exhibits a decline over the course of the work, suggesting that fatigue impacts the diagnostic performance [7]. These issues are further exacerbated by the global shortage of radiologists, which poses a significant challenge to healthcare systems worldwide [8].

One potential approach to address these challenges is the integration of partially or fully automated reporting. In recent years, this has likely contributed to the growing development and clinical testing of artificial intelligence (AI) algorithms designed to support diagnostic processes. The utilization of AI tools for the detection of ICH in non-contrast cCT has demonstrated encouraging outcomes in numerous studies [9–13]. In this context, a salient challenge for radiologists is the issue of trust, as AI algorithms often operate as “black boxes,” rendering their decision processes challenging to comprehend. Consequently, fostering confidence in AI software and ensuring the transparency of automated processes are imperative prerequisites for successful implementation into clinical workflows [14].

The present study was guided by three overarching objectives: First, the performance of an optimized, deep-learning-based AI algorithm for ICH detection in non-contrast cCT scans was evaluated. Second, the objective was to assess the diagnostic performance of the AI in comparison to a conventional radiological report that does not utilize software support to evaluate the potential benefits of AI-supported reporting. Third, the software-generated confidence score was analyzed to identify an optimal cutoff to improve diagnostic quality.

## Methods

### Study cohort

In this retrospective, single-center study, all patients who underwent non-contrast cCT exams (with or without cervical spine) at our institution between April 2020 and April 2022 were consecutively enrolled. The exclusion criteria included patients under 18 years of age, repeated exams of the same patient within less than 6 h, and scans not performed on the standard scanner (Somatom Force, Siemens Healthineers AG). Following the application of these criteria, a total of 2960 cCT scans were included in the analysis with the extended prototype (total cohort). It is imperative to acknowledge that a subset of patients underwent repeated examinations during this timeframe.

A subgroup of 996 cCT scans (996/2960, 33.6%) had previously been analyzed by the early prototype and served as a comparison group (comparative cohort). Written radiology reports were exported from electronic health records.

### CT scan protocol

All scans were performed using 120 kVp with automated tube voltage selection and automated tube current modulation. The images were reconstructed using a standard soft tissue kernel (Hr40), a slice thickness and increment of 1.0 mm and iterative reconstruction strength level 3 (ADMIRE, Siemens). This process yielded image stacks comprising approximately 180 slices per case.

### AI software

Siemens Healthineers developed an algorithm for the detection of intracranial hemorrhages. In this study, two prototype versions of this algorithm are compared: (1) Version 1 is an early prototype that provides the global presence/absence of ICH and its subtypes. A refined variant of the early prototype resulted in the product syngo.CT Brain Hemorrhage VB60. The output of this algorithm is limited to a single image, accompanied by a graphic indicating “processing completed” for ICH-negative scans and “suspected bleeding” for ICH-positive cCT scans. (2) Version 2 contains the triage functionality of syngo.CT Brain Hemorrhage VB60, but has been extended to provide additional prototype outputs. These outputs include a case-level subtype classification, an ICH segmentation mask, and the ICH confidence score.

Both prototypes share the same algorithm pipeline: Based on three-dimensional (3D) neural networks with a Dense-U-Net architecture, an artificial intelligence pipeline for automated non-contrast head CT processing was developed. The process is comprised of four distinct phases. Initially, a pre-processing of the input data is conducted: Five primary anatomical landmarks (bregma, crista galli, external occipital protuberance, left and right orbital bones) are identified, and the brain orientation along the midsagittal plane is estimated to minimize variability in the head position. The identification of landmarks is achieved through the training of an artificial intelligence employing multi-scale deep reinforcement learning [15]. Subsequently, the brain is extracted, and prominent features (e.g., the skull) that could impede the detection process are excluded. To accomplish this objective, an image-to-image convolutional network that has been trained with deep supervision and adversarial perturbations is employed [16]. Second, the presence or absence of hemorrhage is determined using a set of dense neural networks that extract features from both axial and coronal reformats. In the absence of suspected ICH, the algorithm halts and yields a case-level negative response as well as an empty segmentation mask. In the event of suspected ICH, the hemorrhage subtypes are detected on a case-level basis. The architecture of the subtype case-level detection is equivalent to the ICH case-level detection. Finally, the hyperintensities are segmented and annotated according to their location. The segmentation module is composed of a neural network that receives the pre-processed volumes and provides the presence of an acute density at five anatomic regions.

**Fig. 1:**
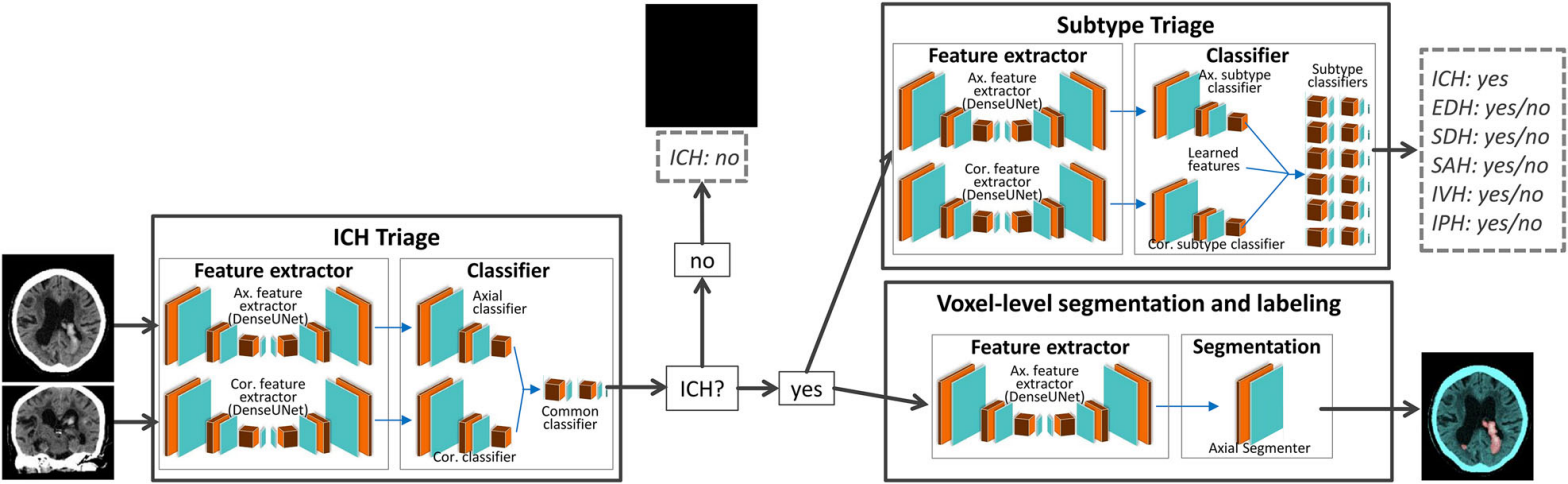
Architecture of the investigated AI algorithm. The AI algorithm processes axial (non-isometric voxels) or coronal (isometric voxels) slices from non-contrast cranial CT scans. After the attenuation of cranial bone and calcifications, hyperdensities are detected by a feature extractor. If no ICH is suspected, the algorithm stops and provides a negative response and an empty segmentation mask. In case of suspected ICH, the hemorrhage subtypes are analyzed, and the hyperintensities are segmented and labeled according to their location. ICH, intracranial hemorrhage; EDH, epidural hemorrhage; SDH, subdural hemorrhage; SAH, subarachnoid hemorrhage; IVH, intraventricular hemorrhage; IPH, intraparenchymal hemorrhage

The detection and segmentation pipeline is illustrated in Fig. 1. The network was trained end-to-end with case- and voxel-level supervision for ICH and ICH subtypes to provide a global absence/presence of ICH and the subtypes, as well as the segmentation mask with subtype labels.

In comparison with the prototype version 1, the extended version of syngo.CT Brain Hemorrhage VB60 (version 2) has been augmented with additional training cases. A total of approximately 28,000 non-contrast head CT series were utilized for training, derived from six clinical sites across the United States, Canada, and India. The characteristics of the training data are as follows: The proportion of ICH-positive subjects was found to be 35%, with an equal distribution of male and female patients. The slice thicknesses ranged from 1 mm to 5 mm, with the acquisition performed using scanners manufactured by Siemens (45%) and other manufacturers (55%).

Furthermore, version 2 of the model was trained with voxel-level subtype annotations in addition to case-level labels. The confidence scores for each prediction are calculated using two approaches [12]: a calibrated classifier score, which reflects how close the prediction is to the decision threshold, and a Dempster–Shafer–based score, which accounts for the model’s internal uncertainty. These values are then aggregated into a composite metric ranging from 0 (indicating high confidence) to 1 (indicating low confidence). This approach facilitates the prioritization of cases and identification of uncertain findings. These values are combined to calculate the final confidence score, which ranges from 50% (low confidence) to 100% (total confidence).

### Data analysis and ground truth

The ground truth was defined as the finalized written radiology report, initially prepared by a radiology resident and supervised by a board-certified radiologist. In the event of a discrepancy between the report and the AI prediction, a second-look assessment was performed by an additional board-certified radiologist with 6 years of CT experience (P.G.) to establish the final diagnosis.

#### Total cohort

A dichotomous classification (presence vs. absence of ICH) was performed for both the extended prototype and the corresponding radiological reports. In cases identified as ICH-positive, the automated categorization into the five subtypes: subarachnoid hemorrhage (SAH), subdural hemorrhage (SDH), epidural hemorrhage (EDH), intraparenchymal hemorrhage (IPH), and intraventricular hemorrhage (IVH) was analyzed.

#### Comparative cohort

The classification results of the early prototype regarding (1) the presence or absence of ICH and (2) the subtype categorization were documented and compared to both the radiology reports and the corresponding predictions from the extended prototype. Due to the limited number of EDH cases in the comparison group (*n* = 7), this subtype was excluded from the direct comparison.

The confidence scores from the AI predictions by the extended prototype were recorded for each case and subsequently analyzed using thresholds ranging from 55% to 100% in 5% increments. This analysis was conducted to identify an optimal cutoff that maximized accuracy and minimized the number of new false-positive results. All cases with a confidence score below the selected threshold were classified as ICH positive. This ensured that cases deemed uncertain by the AI were flagged for human review. The area under the curve (AUC) was used to assess the diagnostic accuracy of the model. Furthermore, all negative cases that were classified as suspicious by adjusting the confidence level threshold were recorded numerically to measure the additional effort required for human follow-up checks.

### Statistical analysis

The statistical analysis was conducted using specialized software (DATAtab e.U.). Ordinal variables are reported as median and interquartile range, while continuous variables are reported as mean and standard deviation. The results were subjected to a comparison using the Wilcoxon test. For the purpose of determining statistical significance, an alpha level of *p* < 0.05 was established. The diagnostic performance of the AI algorithm was assessed by calculating standard diagnostic metrics, including specificity, sensitivity, accuracy, positive predictive value (PPV), and negative predictive value (NPV).

These statistical indicators were used to evaluate the performance of the extended prototype for detecting ICH in the total cohort and to compare the early prototype and the extended prototype in the comparative cohort. The same approach was used to assess the diagnostic performance for subtype classification in both groups. Examinations initially misclassified as ICH-negative were excluded from the subtype analysis, as the algorithm first determines the presence of ICH and only performs the subtype analysis for cases identified as ICH-positive. The confidence scores provided by the AI algorithm were then analyzed using receiver operating characteristic (ROC) curves by plotting the reciprocal of the specificity (*x*-axis) against the sensitivity (*y*-axis). The objective of the ROC curve analysis was to ascertain the optimal confidence thresholds that would enhance the diagnostic performance of the AI software, particularly with respect to the area under the ROC curve (AUC). A range of thresholds from 55% to 100%, with increments of 5%, was evaluated to ascertain the optimal cutoff value that would maximize the trade-off between sensitivity and specificity.

## Results

### Study cohort

The analysis included a total of 2960 cCT scans from 2486 patients. Among the sample, 1091 participants were female (43.9%) and 1395 were male (56.1%). The median age of the participants was 74 years, with an interquartile range of 23 years. The comparative cohort comprised 901 patients, exhibiting a congruent gender and age distribution. The demographic data is summarized in Table 1. To ensure the reliability of the findings, identical CT image stacks were utilized for all evaluations, thereby mitigating the potential impact of patient habitus and positioning on the results.

**Table 1:** Demographic characteristics of the study collective

	Total cohort	Comparative cohort
Patients *n*	2476	901
Female *n* (%)	1089 (44.0%)	394 (43.7%)
Male *n* (%)	1387 (56.0%)	507 (56.3%)
Age years		
Median, interquartile range	74, 23	74, 23
Minimum|maximum	18|105	18|99

### Detection of ICH—performance of AI versus radiologist in the total cohort

A total of 314 scans were classified as positive for ICH, corresponding to an initial prevalence of 10.6%, as determined by the reviewed radiological reports that serve as ground truth.

**Fig. 2:**
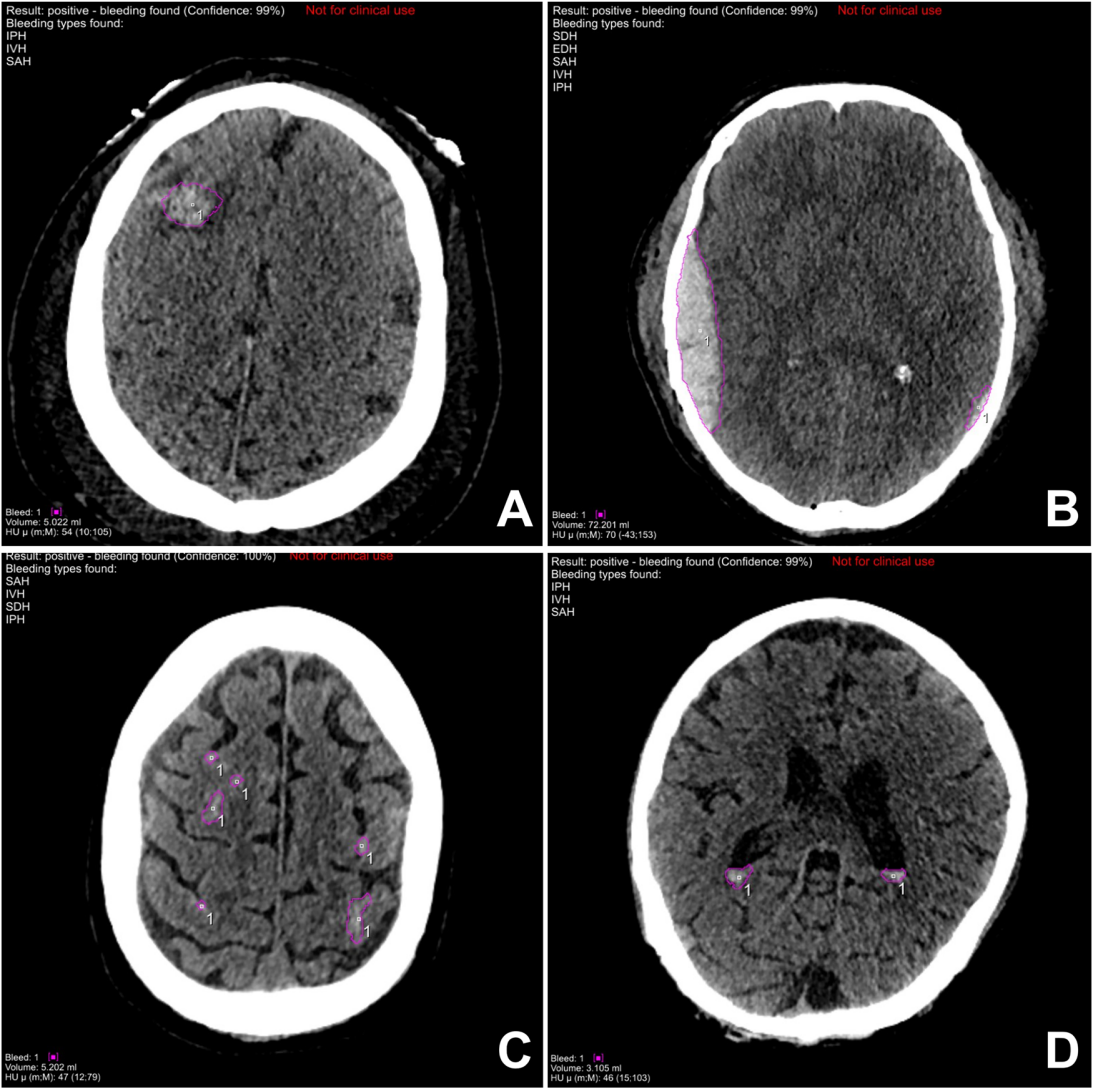
Correct hemorrhage detection with the extended prototype. True positive findings are marked with purple circles. **A** Intraparenchymal hemorrhage. **B** Epidural hematoma. **C** Subarachnoid hemorrhage. **D** Intraventricular hemorrhage

**Fig. 3:**
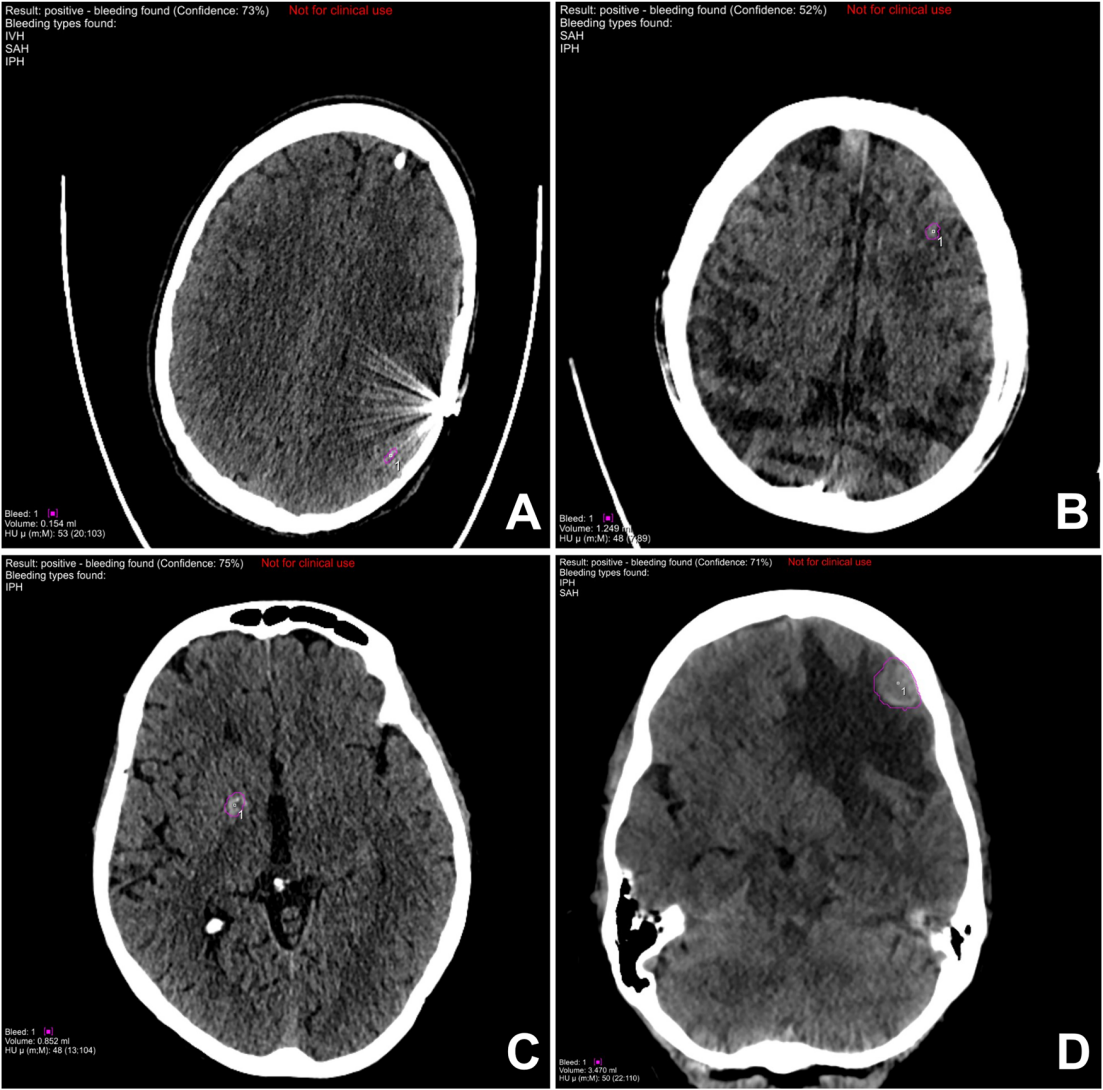
Typical false-positive findings of the extended prototype. False-positive findings are marked with purple circles. **A** Hardening artifacts. **B** Motion artifacts. **C** Parenchymal calcification. **D** Malignancy with perifocal edema

The AI algorithm accurately classified 294 of 2960 cases as ICH-positive and 2545 of 2960 cases as ICH-negative. However, 101 cases were erroneously classified as positive (3.4%), and 20 cases were incorrectly classified as negative (0.7%). The results of this study correspond to a sensitivity of 93.6%, specificity of 96.2%, negative predictive value of 99.2%, and overall accuracy of 95.9%. Table 2 provides a synopsis of the diagnostic performance of the extended prototype. As illustrated in Fig. 2, the AI algorithm has identified a series of true positive findings. Figure 3 presents examples of typical false-positive findings.

**Table 2:** Analysis of automated hemorrhage detection with the extended prototype in a sample of 2960 CT scans in comparison to radiologists

Extended prototype					Radiologists				
TP	TN	FP	FN	∑	TP	TN	FP	FN	∑
294	2545	101	20	2960	299	2643	3	15	2960
Sensitivity		93.6%			Sensitivity		95.2%		
Specificity		96.2%			Specificity		99.9%		
Accuracy		95.9%			Accuracy		99.4%		
NPV		99.2%			NPV		99.4%		
PPV		74.4%			PPV		99.0%		

*NPV* negative predictive value, *PPV* positive predictive value, *TP* true positive, *TN* true negative, *FP* false positive, *FN* false negative

A total of 12 ICH-positive cases were initially missed or not documented by the interpreting radiologists but were correctly detected by the AI algorithm and confirmed as true positives during a second review.

### Detection of ICH—comparison of AI software prototypes in the comparative cohort

The comparative cohort of 996 cases demonstrated a similar incidence of ICH-positive cases with 10.3% (103/996).

**Fig. 4:**
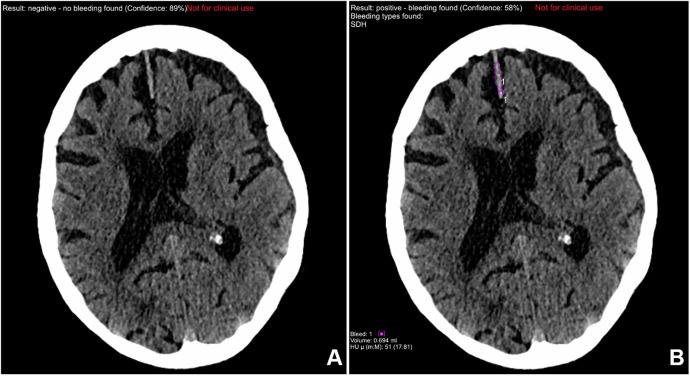
Comparison of subdural hemorrhage detection with the two software prototypes. **A** The early prototype with a missed parafalxin subdural hematoma. **B** The extended prototype with the correct finding (marked with a purple circle)

In this cohort, the extended prototype correctly detected 91 out of 103 ICH-positive cases, resulting in a sensitivity of 88.3%, specificity of 95.1%, and an overall accuracy of 94.4%, as well as an NPV of 98.6%. In contrast, the early prototype detected 80 of the 103 ICH-positive cases, yielding a sensitivity of 77.7%, specificity of 97.5%, and an overall accuracy of 95.5%, as well as an NPV of 97.4%. A comparison of the early prototype to the extended prototype revealed an enhancement in sensitivity and negative predictive value, with eight fewer false-negative results and eleven additional true-positive findings. The comparative results of the two software prototypes are summarized in Table 3. As illustrated in Fig. 4, a notable improvement in the detection of SDH is evident when compared to the initial prototype.

**Table 3:** Intraindividual comparison of automated hemorrhage detection with the two prototypes in a sample of 996 CT scans

Extended prototype					Initial prototype				
TP	TN	FP	FN	∑	TP	TN	FP	FN	∑
91	849	44	12	996	80	871	22	23	996
Sensitivity		88.3%			Sensitivity		77.7%		
Specificity		95.1%			Specificity		97.5%		
Accuracy		94.4%			Accuracy		95.5%		
NPV		98.6%			NPV		97.4%		
PPV		67.4%			PPV		78.4%		

*NPV* negative predictive value, *PPV* positive predictive value, *TP* true positive, *TN* true negative, *FP* false positive, *FN* false negative

### Subtype analysis

#### Total cohort

Subtype analysis was performed for the extended prototype in 294 cases that were classified as true ICH-positive by the AI algorithm. The results of the subgroup classification and the corresponding diagnostic metrics are summarized in Table 4. It is noteworthy that the extended prototype demonstrated superior performance in two subtype categories when compared to the radiologists’ assessment. For SAH, the extended prototype exhibited a sensitivity of 87.1%, correctly identifying 176 out of 202 cases. In comparison, radiologists detected only 158, resulting in a sensitivity of 78.2%. A comparable situation is observed in the context of IVH, wherein the extended prototype detected 69 of 73 cases with IVH (sensitivity 94.5%) in contrast to the 52 cases identified by the radiologists (sensitivity 71.2%).

**Table 4:** Analysis of hemorrhage subtype classification with the extended prototype in a sample of 2960 CT scans

Extended prototype															
SAH				SDH				IPH				IVH			
TP	TN	FP	FN	TP	TN	FP	FN	TP	TN	FP	FN	TP	TN	FP	FN
176	76	36	26	127	95	78	14	131	86	81	16	69	155	86	4
∑ = 202				∑ = 141				∑ = 147				∑ = 73			
Sensitivity		87.1%		Sensitivity		90.1%		Sensitivity		89.1%		Sensitivity		94.5%	
Specificity		67.9%		Specificity		54.9%		Specificity		51.5%		Specificity		64.3%	
Accuracy		80.3%		Accuracy		70.7%		Accuracy		69.1%		Accuracy		71.3%	
NPV		74.5%		NPV		87.2%		NPV		84.3%		NPV		97.5%	
PPV		83.0%		PPV		62.0%		PPV		61.8%		PPV		44.5%	

*SAH* subarachnoid hemorrhage, *SDH* subdural hemorrhage, *IPH* intraparenchymal hemorrhage, *IVH* intraventricular hemorrhage, *NPV* negative predictive value, *PPV* positive predictive value, *TP* true positive, *TN* true negative, *FP* false positive, *FN* false negative

#### Comparative cohort

In the comparative cohort, the extended prototype correctly detected 91 ICH-positive cases, whereas the early prototype detected 80 cases. In order to ensure the comparability of the data and to avoid any potential bias that might be introduced by differing case numbers, the subtype analysis was limited to the 80 cases that were detected by both algorithm prototypes. For SAH, the extended prototype demonstrated a sensitivity of 96.2% by detecting 50 out of 52 cases, while the early prototype exhibited a sensitivity of 84.6% by detecting 44 out of 52 cases. With respect to the issue of IVH, the sensitivity exhibited an increase from 85.0% in the initial prototype to 95.0% in the extended prototype. Consequently, the NPV increased from 93.3% in the initial prototype to 96.8% in the extended prototype. The extended prototype exhibited an enhanced sensitivity of 96.8%, accompanied by an NPV of 95.5%, in comparison to the initial prototype (sensitivity 93.5%, NPV 94.1%). A substantial improvement in the capacity to detect IPH was evident, with the extended prototype demonstrating a sensitivity of 92.9%, as indicated by its correct identification of 39 out of 42 cases. In comparison, the initial prototype exhibited a sensitivity of 54.8%, detecting only 23 out of 42 cases. The results of the subtype analysis for both software prototypes in the comparative cohort are presented in Table 5.

**Table 5:** Intraindividual comparison of hemorrhage subtype classification with the two software prototypes in a sample of 996 CT scans

Extended prototype															
SAH				SDH				IPH				IVH			
TP	TN	FP	FN	TP	TN	FP	FN	TP	TN	FP	FN	TP	TN	FP	FN
50	13	13	2	30	21	26	1	39	10	26	3	19	30	28	1
∑ = 52				∑ = 31				∑ = 42				∑ = 20			
Sensitivity		96.2%		Sensitivity		96.8%		Sensitivity		92.9%		Sensitivity		95.0%	
Specificity		50.0%		Specificity		44.7%		Specificity		27.8%		Specificity		51.7%	
Accuracy		80.8%		Accuracy		65.4%		Accuracy		62.8%		Accuracy		62.8%	
NPV		86.7%		NPV		95.5%		NPV		76.9%		NPV		96.8%	
PPV		79.4%		PPV		96.8%		PPV		60.0%		PPV		40.4%	

Initial prototype															
SAH				SDH				IPH				IVH			
TP	TN	FP	FN	TP	TN	FP	FN	TP	TN	FP	FN	TP	TN	FP	FN
44	17	9	8	29	32	15	2	23	27	9	19	17	42	16	3
∑ = 52				∑ = 31				∑ = 42				∑ = 20			
Sensitivity		84.6%		Sensitivity		93.5%		Sensitivity		54.8%		Sensitivity		85.0%	
Specificity		65.4%		Specificity		68.1%		Specificity		75.0%		Specificity		72.4%	
Accuracy		78.2%		Accuracy		78.2%		Accuracy		64.1%		Accuracy		75.6%	
NPV		68.0%		NPV		94.1%		NPV		58.7%		NPV		93.3%	
PPV		83.0%		PPV		65.9%		PPV		71.9%		PPV		51.5%	

*SAH* subarachnoid hemorrhage, *SDH* subdural hemorrhage, *IPH* intraparenchymal hemorrhage, *IVH* intraventricular hemorrhage, *NPV* negative predictive value, *PPV* positive predictive value, *TP* true positive, *TN* true negative, *FP* false positive, *FN* false negative

### Confidence score—threshold

**Fig. 5:**
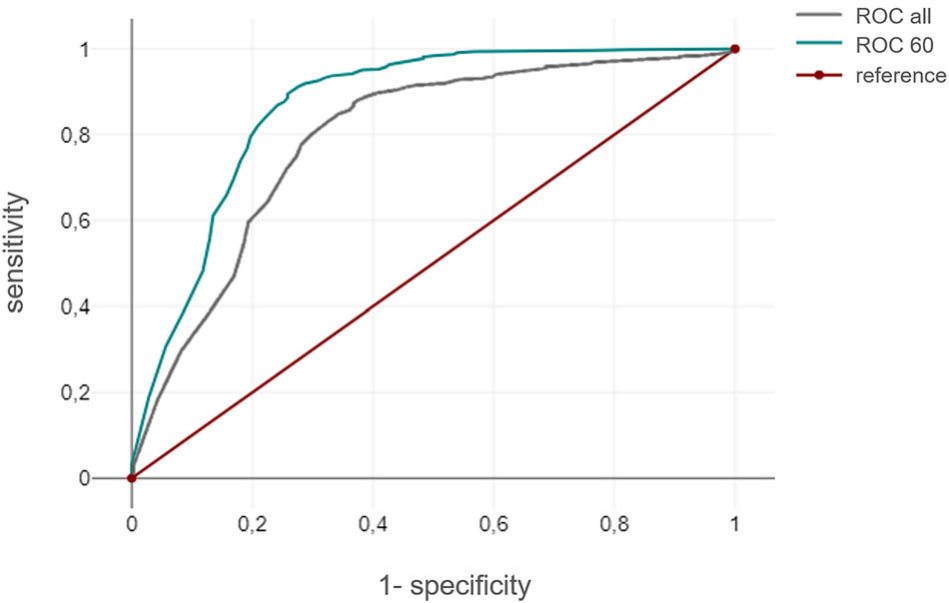
Receiver operating characteristic (ROC) curve analysis of intracranial hemorrhage detection without threshold (ROC all) and with 60% threshold (ROC 60)

The ROC curve analysis demonstrated a significant increase in the AUC when establishing a confidence interval threshold of 60%. The AUC of the overall dataset was 0.797, while the AUC of a confidence score > 60% was 0.865 (*p* = 0.018; Fig. 5). This adjustment has been to increase the precision of positive findings by 7 to 299, while concurrently reducing the number of false negative findings by 3 to 16. However, according to the principle that AI results with a confidence score < 60% are considered suspicious, 2.46% more cCT examinations would have to undergo radiological review, which corresponds to 73 scans in our cohort.

## Discussion

The prompt and accurate diagnosis of intracranial hemorrhages (ICH) on CT is of high clinical relevance. Maintaining diagnostic quality amidst rising case volumes and increasing workload during both day and night shifts remains a key challenge. The objective of this retrospective study was therefore to evaluate the performance of an extended prototype of a deep-learning-based artificial intelligence algorithm for ICH detection in non-contrast cCT scans. The study compared the performance of an initial prototype to the latest algorithm version as well as routine radiological reports. The efficacy of the extended prototype in enhancing diagnostic performance was demonstrated, exhibiting results comparable to those of a human reader. In a similar vein, the subgroup classification of hemorrhage underwent enhancement with the extended prototype, leading to a more precise categorization in comparison to the radiologists’ reports.

The performance of the examined AI algorithm was analogous to that of previously documented AI applications for ICH detection. The precision parameters generally exhibited a similar range and demonstrated a strong correlation with the results obtained by human readers in our study. The majority of AI applications report sensitivity values exceeding 90.0% for intracranial hemorrhage [11, 12]. A number of groups have reported remarkably high diagnostic values. For instance, Kiefer et al reported a sensitivity of 98.1% and an NPV of 99.7% [11]. However, the relatively small sample size in their study, comprising only 432 and 52 ICH-positive cases, may limit the generalizability of their results. Kang et al reported a comparable performance, with sensitivity values of 98.7% in a cohort of 1370 cases. However, the specificity was only 88.5%, indicating an optimization for detecting ICH rather than ruling out, as intended in the examined algorithm [17]. In contrast, Tommaso et al reported a lower performance, exhibiting a sensitivity of 90.4%, a specificity of 94.9%, and an NPV of 70.2% suggesting a higher ratio of missed cases [18].

The process of subtyping the identified intracranial hemorrhages remains a challenging task. The extended algorithm yielded satisfactory sensitivities (96.2% and 92.9%, respectively) for SAH and IPH, respectively. However, its specificity could be improved. It is noteworthy that other developers have reported varying outcomes in regard to classification accuracy, which appear to vary according to the subtype, presumably as a consequence of the distribution present within the test dataset [19–21].

Beyond achieving diagnostic quality that is comparable to or even surpasses that of a human radiologist, the trustworthiness and interpretability of AI results are particularly relevant for successful integration and acceptance into clinical practice [14]. The NPV can function as an objective indicator of trustworthiness: Conversely, an elevated NPV signifies a heightened probability that a negative result will indeed be negative, thereby mitigating the risk of overlooked pivotal findings. The high NPV of the extended prototype (99.2%) underscores the high reliability of its negative predictions, indicating that the AI can effectively preselect cases and flag almost all conspicuous findings with a low false negative rate of 7 cases per 1000. This result suggests potential for workload reduction, particularly during periods of limited staffing, and to help prioritize clinically relevant cases.

The extended prototype detected 12 additional ICH cases in the total cohort that had initially gone undetected by radiologists. This underscores the efficacy of concurrent AI integration during routine reporting. While the sustainability of this advantage in the context of AI utilized solely for pre-selection to mitigate reporting obligations remains a subject of discourse, the concomitant application of AI alongside radiologists may augment diagnostic confidence. In instances of conflicting interpretations, this dual reading approach fosters critical analysis and, when necessary, the rectification of misinterpretations. Research has demonstrated that diagnostic performance tends to decline outside of standard working hours [22]. Furthermore, a prior investigation by Gruschwitz et al demonstrated the potential for expediting the diagnostic process through the utilization of an AI algorithm [13].

The performance evaluation of the current algorithm reveals that specificity and overall accuracy exhibit a tendency to be lower in comparison to sensitivity and NPV when contrasted with the initial version. Given that the AI algorithm under investigation was principally designed as a triage instrument and not as a definitive diagnostic system, sensitivity and, in particular, NPV were deliberately maximized during the development phase. The objective of the study was to minimize the number of patients with actual intracranial hemorrhage who were incorrectly classified as normal. The current version of the algorithm therefore delivers an optimized NPV (99.2%) with the disadvantage of an inevitable increase in the false-positive rate and thus a decrease in the measured specificity and overall accuracy. This is because the goal is not primarily a perfect classification model, but rather a reliable exclusion tool in the triage workflow. A similar performance profile has been reported in other comparable studies in clinical settings, as evidenced by Seyam et al These studies have indicated a notable similarity in the performance values, with the NPV ranging from approximately 97.8%, seemingly consistent with the same underlying intention [10]. This correlation has been corroborated through meta-analyses, which provide a quantitative assessment of the literature on the subject. AI models have been shown to demonstrate high sensitivity and NPV in the detection and exclusion of ICH. However, specificity is more variable and is influenced by the triage-oriented orientation of the algorithms [23].

In addition, concerted efforts have been made to improve the transparency and traceability of AI decision-making processes. The AI utilized in this study generates two distinct types of output: first, it emphasizes all areas identified as bleeding through the use of color overlays, and second, the AI provides a confidence score, which serves as an indication of the certainty of its prediction. The utilization of color-coded visualizations enables a verification process that is both time-efficient and targeted, thereby ensuring the alignment of the AI’s prediction with the user’s own interpretation. As shown in Fig. 3, the AI algorithm has been shown to detect common false positives, including motion artifacts, beam-hardening artifacts, and calcifications. These artifacts are also well-known sources of error in other algorithms, which can be swiftly eliminated by the reading radiologist [11, 12]. It is noteworthy that the mean patient age in our cohort (70 years) was relatively high, which may have contributed to a higher prevalence of calcifications, as previously described by Saade et al [24]. Another relatively recent approach involves the generation of AI confidence scores. In accordance with Gibson et al, a strong correlation was observed between accurate AI findings and high confidence scores [12]. Therefore, it can be posited that the confidence metric may be utilized as an auxiliary criterion for reliability, and it is conceivable that the diagnostic accuracy may be augmented by establishing a threshold.

In many cases, the assignment of ICH to a specific subtype is irrelevant for further procedures. However, in certain instances, further elucidation is necessary to ensure a comprehensive understanding. For instance, the detection and classification of SAH is of particular relevance, as atraumatic SAH is often associated with aneurysm rupture and typically necessitates additional CT angiography [25]. This principle similarly applies to atypical parenchymal hemorrhages, necessitating further investigation. Accurate subtype classification has the potential to identify such findings in a timely manner, enabling prompt follow-up examinations. Consequently, the integration of AI with healthcare systems has the potential to enhance operational efficiency, reduce unnecessary delays, and optimize patient transfers, thereby facilitating more expeditious diagnosis and treatment. The extended prototype demonstrated enhanced sensitivity and NPV for four out of five subtypes of ICH compared to the early prototype, indicating potential improvements in subtype assignment. Furthermore, the AI exhibits superior performance in detecting SAH and IVH, indicating that the detection of smaller bleeding volumes can also be reliably achieved. In the context of SAH detection, the investigated algorithm demonstrated a comparable performance to a similar algorithm developed by another research group in a clinical cohort [12] and exhibited superior sensitivity compared to the algorithm from Seyam et al (sensitivity = 80.0%) [10].

### Limitations

Our single-center study, which employed a retrospective design, encompasses one of the most extensive datasets of 2960 cCTs investigated for the purpose of automated ICH detection. However, it is important to note that the study is not without some limitations. First, defining the radiology report as ground truth may introduce bias, particularly in cases where both the radiologist and the AI algorithm failed to detect ICH. Second, the second-look review of discrepant findings was performed by one radiologist, who was not blinded to the results of both artificial intelligence and the reading radiologists, which could also introduce interpretive bias. Furthermore, a definitive evaluation of the diagnostic accuracy of the radiological findings with and without AI support was not conducted. This issue must be addressed in future research.

The single-vendor nature of this study might limit the generalizability of findings; however, to date, the investigated AI tool is not currently available across different platforms. It is noteworthy that the present study incorporated exclusively CT images obtained with a single scanner model and a standardized examination protocol, thereby yielding a remarkably homogeneous test dataset. While this consistency ensures internal validity, it may also lead to an overestimation of the model’s performance in more heterogeneous clinical environments. However, given that this was an all-comers study with consecutive enrollment, the heterogeneity of the patient cohort was ensured. Finally, the integration of the AI tool into the clinical workflow was not evaluated, nor was its use for pre-selecting ICH-positive cases and the potential acceleration of the reporting process that would result from its use.

### Future direction

To validate our results, a prospective multicenter clinical trial involving multiple CT systems from different vendors is needed to assess the generalizability and robustness of the AI algorithm in diverse clinical settings. Similarly, the confidence level adaptation for other diagnostic sites must be reevaluated.

## Conclusion

The improved AI algorithm achieves high sensitivity, diagnostic accuracy, and negative predictive value. Combining AI-driven analysis with the expertise of radiologists may improve the overall performance, reducing the number of missed intracranial hemorrhages and resulting in faster, more accurate diagnosis.

## Data Availability

The datasets generated and/or analyzed during the current study are not publicly available but are available from the corresponding author on reasonable request.

## Abbreviations

AI: Artificial intelligence algorithm
AUC: Area under the curve
cCT: Cranial computed tomography
EDH: Epidural hemorrhage
ICH: Intracranial hemorrhage
IPH: Intraparenchymal hemorrhage
IVH: Intraventricular hemorrhage
NPV: Negative predictive value
PPV: Positive predictive value
ROC: Receiver operating characteristic
SAH: Subarachnoid hemorrhage
SDH: Subdural hemorrhage
